# Another Answer to Dr. Allen’s Protest

**Published:** 1890-07

**Authors:** G. W. Sherbino

**Affiliations:** Abilene, Texas


### ANOTHER ANSWER TO DR. ALLEN'S PROTEST.

EDITORS OF THE HOMŒOPATHIC PHYSICIAN:—In reading Dr. Allen's Protest, I come to a portion of it in which I cannot agree with him. He classes *me* with the allopaths, because I use high potencies and report clinical symptoms, thus filling the journal full of rubbish and trash that is not worth the paper upon which it is printed. Of course the shoe fits me and I want my say about the matter. A proving first upon the healthy is always desirable. This is Hahnemann's teaching. But if you throw out valuable clinical symptoms that have been obtained from the experience at the bedside you will throw away a great deal of the most valuable part of our materia medica.

I cannot see how the Doctor classes clinical symptoms with high potency allopathy. I never knew that the allopath ever prescribed upon clinical symptoms; their prescriptions are always based upon some pathological idea.

I am called to treat a sick patient and I take the totality of the symptoms. I find the remedy. I find also other symptoms not mentioned in the materia medica that the patient has. I prescribe the indicated remedy from the most prominent and peculiar symptoms. They are cured, also the rest of the symptoms which are clinical. Now I have learned something new about this remedy from clinical observation. If I had no observation I would not pay any attention to these symptoms, which are not recorded in the materia medica. Having observation, however, I notice these symptoms and put them down for further verification in other cases and thus determine if they are true and reliable. I think that the suppressing clinical symptoms that are thus found reliable would be a great wrong.

The value of the remedy is determined by provings upon the healthy and by clinical symptoms that have been well verified until a further proving may develop the true picture of the drug.

A confirmed clinical symptom is as reliable to prescribe from as a symptom from a proving. The drug would not cure if not able to produce this symptom upon the healthy.

Hence it is not allopathic empiricism, even though it has been derived from observation or experience with a sick patient. It is a practical observation obtained during the process of healing, and not from any pathological supposition.

Take an instance: *The child gets upon* its hands and knees at night and sleeps that way. This symptom can't be found in the materia medica, yet it is a well verified symptom, belongs to only one remedy, and that one always cures this condition. I have verified it hundreds of times; this is only one symptom, and other symptoms of the remedy are present, but one symptom is valuable when no more can be obtained from the patient.

I give here an empirical allopathic, prophylactic, cholera prophylactic.

℞ Magnesia Sulphite, . . . . . . . . . . 2 dr.
Sulphurous Acid, . . . . . . . . . . . 16 dr.
Water, . . . . . . . . . . . . . . . . . 16 dr.
Tincture Capsicum, . . . . . . . . . . 4 dr.

M. Dissolve perfectly.
Sig. Teaspoonful night and morning.

This is what the doctor classifies with symptoms cured by high potencies. I cannot see any analogy whatever. He may not, however, have the same focus; this probably is where the fault lies, we don't see alike.

I have great respect for Dr. Allen. I have all the books he has ever published and I know he has done a great work for Homœopathy in his great materia medica; but upon the value of clinical symptoms I will have to differ with him. I would rather have them to prescribe from than the ℞ of cholera prophylactic. A high potency will not suppress or kill as quickly as a scientific (?) mixture of strong drugs.

I have been helped out many a time when I would have been obliged to use a palliative but for a well-known clinical symptom.

G. W. Sherbino.

Abilene, Texas, May 2d.

